# Global child and adolescent mental health: The orphan of development assistance for health

**DOI:** 10.1371/journal.pmed.1002524

**Published:** 2018-03-09

**Authors:** Chunling Lu, Zhihui Li, Vikram Patel

**Affiliations:** 1 Division of Global Health Equity, Brigham and Women's Hospital, Boston, Massachusetts, United States of America; 2 Department of Global Health and Social Medicine, Harvard Medical School, Boston, Massachusetts, United States of America; 3 Department of Science and Technology-National Research Foundation (DST-NRF) Center of Excellence in Human Development, University of Witwatersrand, Johannesburg, South Africa; 4 Department of Global Health and Population, Harvard T.H. Chan School of Public Health, Boston, Massachusetts, United States of America

## Abstract

In an analysis of data from the Creditor Reporting System, Chunling Lu and colleagues investigate the level of development assistance from high-income countries towards child and adolescent mental health in low- and middle-income countries.

**Summary points**

- One-quarter of disability-adjusted life years (DALYs) for mental disorders and substance abuse is borne by those 24 years old or younger, the age group that accounted for more than 40% of the world population. Using the aid activities database from the Creditor Reporting System (CRS), we estimated the level of development assistance for child and adolescent mental health (DAMH_CA) in 132 developing countries between 2007 and 2015.
- The total amount of DAMH_CA with a primary target on child and adolescent mental health was US$190.3 million over the 8 years, accounting for 12.5% of total development assistance for mental health (DAMH) and 0.1% of development assistance for health (DAH).
- The largest investments in DAMH_CA over this 8-year period were disbursed to the humanitarian assistance sector for children and adolescents in disasters or conflicts (US$77.2 million [41% of DAMH_CA]), followed by the sector of government and civil services (US$58.6 million [31%]), the health sector (US$38.0 million [20%]), and the education sector (US$15.6 million [8%]).
- Donors invested little in child and adolescent mental health, in both absolute amount and fraction.
- The donor community should substantially increase DAMH_CA to establish and enhance the capacity for delivering mental health care to this demographic group.

## Background

More than 40% of the world population is 24 years old or younger, the vast majority of whom live in low- and lower middle–income countries [1]. Globally, a quarter of disability-adjusted life years (DALYs) for mental disorders and substance abuse is borne by this age group [2], and about 75% of mental disorders diagnosed in adulthood have their onset before the age of 24 years [3]. Most children and young people in developing countries, however, do not have access to mental health care.

Lack of financial commitment is amongst the major barriers for improving access to mental health interventions in developing countries. Unsurprisingly, the least resourced regions and countries in the world rely heavily on development assistance, typically from high-income countries or foundations, to support the health sector. Our previous study on development assistance for mental health (DAMH) demonstrated that DAMH remained low both in absolute terms and as a proportion of development assistance for health (DAH) between 2007 and 2013 [4].

This analysis extends our previous analysis by investigating development assistance for child (below age 10) and adolescent (between age 10 and 24) mental health (DAMH_CA). We tracked the level of DAMH_CA in 132 countries between 2007 and 2015 (**S1 Table**).

## Estimating DAMH_CA between 2007 and 2015

### Data sources

We used the Organization for Economic Co-operation and Development (OECD) Creditor Reporting System (CRS) aid activity database [5], which was downloaded in May 2017. The CRS database is publicly accessible and provides information on aid activities reported directly by the governments of the 26 members of the Development Assistance Committee (DAC), multilateral organizations (e.g., World Bank), global health initiatives (e.g., GAVI, the Vaccine Alliance), non-DAC countries (e.g., United Arab Emirates), and private donors (the Bill & Melinda Gates Foundation [BMGF]) [5,6]. See **S1 Text** for more details on data sources. Our analysis included 132 countries (**S1 Table**) founded by 44 donors.

### Identifying projects on child and adolescent mental health

We defined DAMH_CA as aid disbursed to projects that either primarily or partially targeted the promotion of mental health or the prevention and treatment of mental disorders for children or adolescents. We adopted a multi-sectoral perspective [4,7] and included mental health projects in non-health sectors such as education, government and civil services, and humanitarian assistance.

CRS data do not have a variable to indicate mental health projects for children and adolescents but has three variables (project title, short description, and long description) that allowed us to identify such projects with a list of key words (**S2 Table**) used in the previous studies [4,8]. Key word searches were performed for projects in the sectors listed in **Table 1**. The definition of these sectors in the CRS can be found in **S1 Box**. Details on identification strategy are presented in **S1 Text**.

**Table 1:** Sectors for identifying DAMH_CA projects: Frequency and the two most common themes.

Sectors	Projects with the primary purpose on DAMH_CA (lower bound), *N* = 1,384		Projects primarily or partially on DAMH_CA (upper bound), *N* = 4,009	
	Frequency (Percentage) of Total Projects	Two most common themes	Frequency (Percentage) of Total Projects,	Two most common themes
**Education**				
**Basic Education**	65 (4.70%)	Establish psycho-pedagogical research center for special educational needs; Educate and train students with mental disabilities	423 (10.55%)	Help teenagers grow and enhance their mental and physical health; Increase parents' knowledge of early childhood development, including cognitive, socio-emotional, linguistic, and physical aspects
**Secondary Education**	16 (4.70%)	Provide special assistance to traumatized children; Provide support to young children to treat or prevent traumatizing	92 (2.29%)	Emotional service for children, along with education, effort, medical service, and cultural exchange; Raise awareness of children's reaction to violence and war, prevent/minimize the long-term effects of trauma
**Postsecondary Education**	22 (1.59%)	Project targeting disruptive behavior and attention disorders of childhood and adolescence; Provide primary care for youth with mental illness	39 (0.97%)	Improve school performance to favor the harmonic physical and psychological development of disadvantaged children; Support public institutions to provide youth with appropriate psychological and social expertise
**Education, Level Unspecified**	36 (2.60%)	Construction of a center for children with autism; Psychological intervention and development project for poor university students	148 (3.69%)	Provide activities to help enhance youths’ mental and physical health; Provide school-based interventions to improve child cognitive and psychosocial development and physical and nutritional health and increase school enrollment rate
**Health**				
**General Health**	225 (16.26%)	Improving psychological healthcare for children; Address negative effects of alcohol and drug use among children and youth	384 (9.58%)	Provide treatment and psychological support for HIV/AIDS patients; Improve physical, nutritional, and psychological services for preschool-aged children
**Basic Health**	88 (6.36%)	Provide psychosocial care for young children; Support a therapy and support center for children with autism	338 (8.43%)	Improve growth and development of children under 6 years old in terms of nutrition, health, psychosocial, and cognitive aspects; Seek to reach children who have both physical and mental disabilities
**Population and Reproductive Health**	35 (2.53%)	Monitor and promote psychosocial care among young children; Provide psychosocial care for children and orphans living with chronic illness	497 (12.40%)	Address the HIV- and AIDS-related needs of high-risk populations, including drug users, sex workers, and orphans; Provide psychosocial and medical support for people to live with HIV/AIDS
**Government and Civil Service**				
**Government and Civil Society**	149 (10.77%)	Control tobacco and alcohol use among children and young people; Improve well-being of children and youth traumatized by displacement and war	482 (12.02%)	Address educational and psychosocial impact of armed conflict, build child protection network, promote rights-based work; Improve physical and mental health of women victims of domestic violence and their children
**Conflict, Peace, and Security**	44 (3.18%)	Improve psychological healthcare for children; Ensure psychological and social support in reintegration in home communities	126 (3.14%)	Provide interventions (psychosocial and medical, and vocational training) for women and child soldiers; Provide jobs, training, funding for income generation and offer remedial schooling, trauma counseling, and family reintegration
**Other Social Infrastructure and Services**				
**Other Social Infrastructure and Services**	404 (29.19%)	Establish a specialized resource center for preschool- and school-age autism service organizations; Raise awareness of the effects of drug abuse among youths	709 (17.69%)	Improve education and work performance, address delays in children’s cognitive development; Provide comprehensive care for children and adolescents for the prevention of antisocial behavior, violence, improper use of drugs and alcohol, and HIV/AIDS
**Humanitarian Aid**				
**Emergency Response**	252 (18.21%)	Provide psychological diagnostics and rehabilitation; Provide psychosocial assistance to earthquake-affected children	585 (14.59%)	Establish children-friendly spaces and provide psychosocial support for children; Provide life skills education and psychosocial support for conflict-affected children and adolescents
**Reconstruction Relief and Rehabilitation**	17 (1.23%)	Provide support and mental health enhancement for children and families; Provide care for traumatized children and adolescents	29 (0.72%)	Provide physical, psychological, and social support for Syrian refugee children and families; Establish a multifunctional social center to provide social services, including hairdresser services and psychosocial care and consultation for children
**Disaster Prevention and Preparedness**	5 (0.36%)	Provide prevention of post-traumatic disorders for children and adolescents; Provide psychosocial interventions for children	15 (0.37%)	Teach communities how to decrease vulnerability and physical and spiritual trauma; Give access to quality basic education for post-earthquake children, along with psychosocial and pedagogical support
**Other Multisector**				
**Other Multisector**	21 (1.52%)	Establish residential center for psychosocially high-risk children; Help children overcome trauma through psychological and social recovery actions	91 (2.27%)	Community-based initiatives to treat depression and provide AIDS prevention for girls; Enable women and girls to recover their self-esteem, to know their rights, to reintegrate them socially, and to improve their living conditions
**Unallocated/Unspecified**				
**Unallocated/Unspecified**	5 (0.36%)	Provide psychosocial support for child refugees; Provide community-based protection services and psychosocial support for children	51 (1.27%)	Provide basic physical care, palliative care, psychosocial support, and counseling, with a particular focus/concern for orphans and vulnerable children; Increase parents’ knowledge in cognitive, socio-emotional, linguistic, and physical aspects of early childhood development

Abbreviation: DAMH_CA, development assistance for child and adolescent mental health.

### Analyzing DAMH_CA

We followed the approach from an earlier study [7] and constructed two sets of estimates: one including the full disbursements of multi-target projects (the upper bound of estimated aid for DAMH_CA, presented in the Supporting information and the other (the lower bound of estimated aid for DAMH_CA, presented in the text), which included only projects with a primary target of promoting mental health for children and adolescents. We used actual disbursements (grants and loans) rather than commitments to estimate DAMH_CA. The time frame allowed us to both track changes in DAMH_CA since *The Lancet* published its first series on mental health and adolescent health [9,10] and to avoid the issue of missing disbursement data in the CRS [6].

We identified the number of projects in each subsector and provided examples of the two most commonly funded themes in the subsector. We estimated levels and changes of DAMH_CA, for each country as well as for the sum of all countries, between 2007 and 2015 in both total and per capita spending, with their upper and lower bounds. We tracked changes in DAMH_CA by sectors, donors, channels of aid delivery (implementing agency), and countries between 2007 and 2015. For projects with missing channels (516 projects, cumulatively [13%]), we assumed they were delivered by donors and replaced the channels according to donor categories. When conducting estimations at the country level, we excluded projects not allocable to a specific country.

We paid special attention to DAMH_CA that targeted suicide and the five types of mental disorders that are the leading causes for disability or death among the children and adolescents group: (1) anxiety, (2) autism, (3) depression, (4) substance abuse, and (5) trauma [11,12].

All disbursements are in 2013 US dollars (USDs). STATA 14 was used in analysis.

## Themes supported by DAMH_CA

We identified 1,384 DAMH_CA projects with primary targets on child and adolescent mental health (lower bound). Among them, 404 were allocated to “Other Social Infrastructure and Services,” and the most common themes were in providing special care for children with autism and raising awareness of drug abuse among youths. There were 252 projects for “Emergency Response,” with the most common themes being providing psychosocial assistance and care to children in disasters or conflict areas. There were 225 projects for “General Health,” with the most common themes being improving psychological healthcare for children and addressing alcohol and drug use among adolescents. There were 149 projects for “Government and Civil Society,” with the most common themes being controlling tobacco and alcohol use and improving well-being for children and youth affected by wars (**Table 1**).

## Absolute and relative DAMH_CA, lower-bound estimates

**Fig 1:**
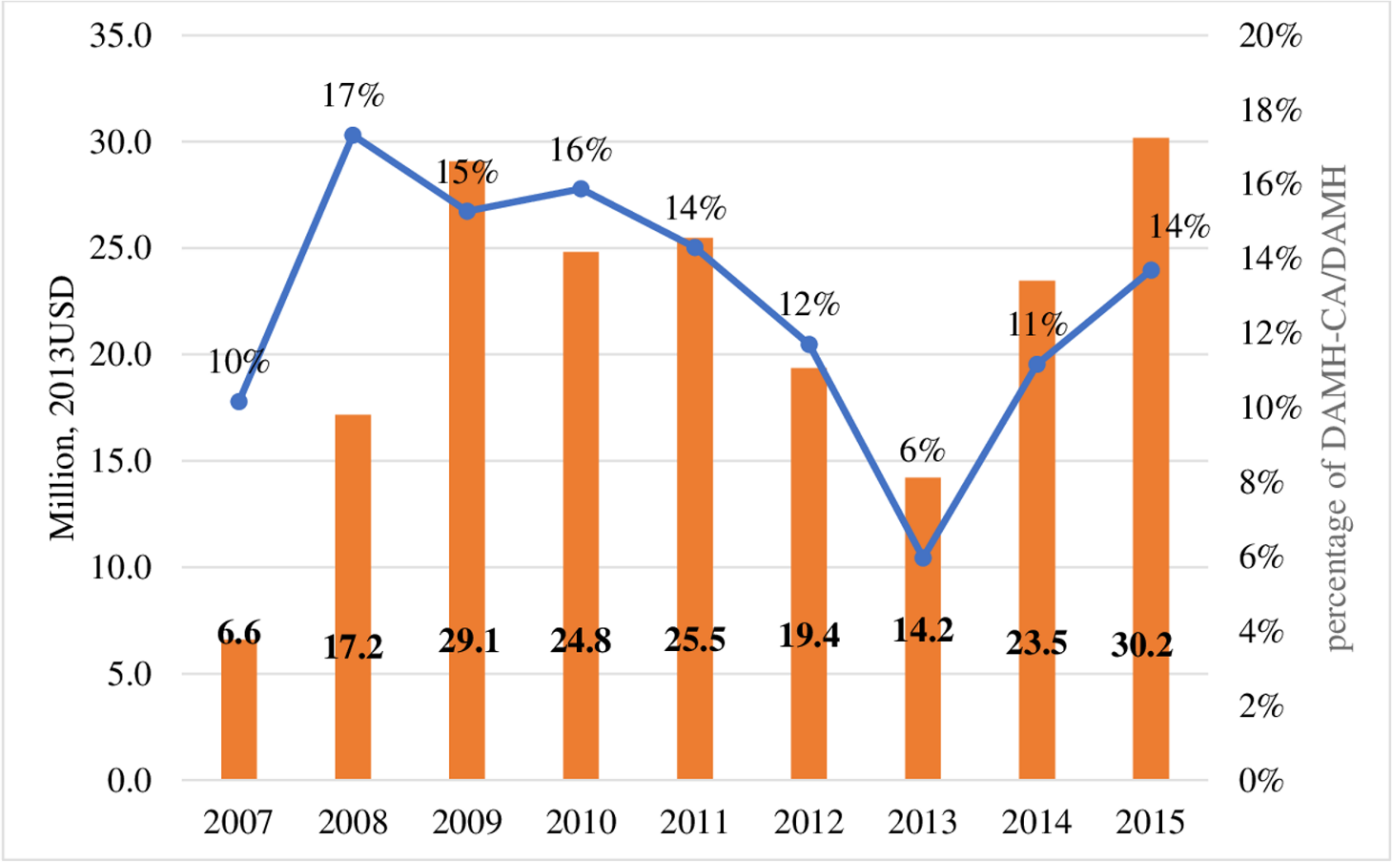
DAMH_CA by year (million, 2013 USD) and DAMH_CA disbursements as percentages of total DAMH, lower-bound estimates. DAMH, development assistance for mental health; DAMH_CA, development assistance for child and adolescent mental health, USD, US dollar.

Between 2007 and 2015, a total of US$190.3 million was disbursed to projects with the primary purpose of improving the mental health of children and adolescents, accounting for 12.5% of DAMH and 0.10% of the total DAH disbursed over this period. The DAMH_CA increased from US$6.6 million in 2007 to US$30.2 million in 2015, with fluctuations over time. The percentages of DAMH_CA in total DAMH increased from 10% in 2007 to 17% in 2008 and dropped to 6% in 2013. In 2015, 14% of DAMH was for child and adolescent mental health (**Fig 1**).

## Sectors receiving DAMH_CA, lower-bound estimates

**Fig 2:**
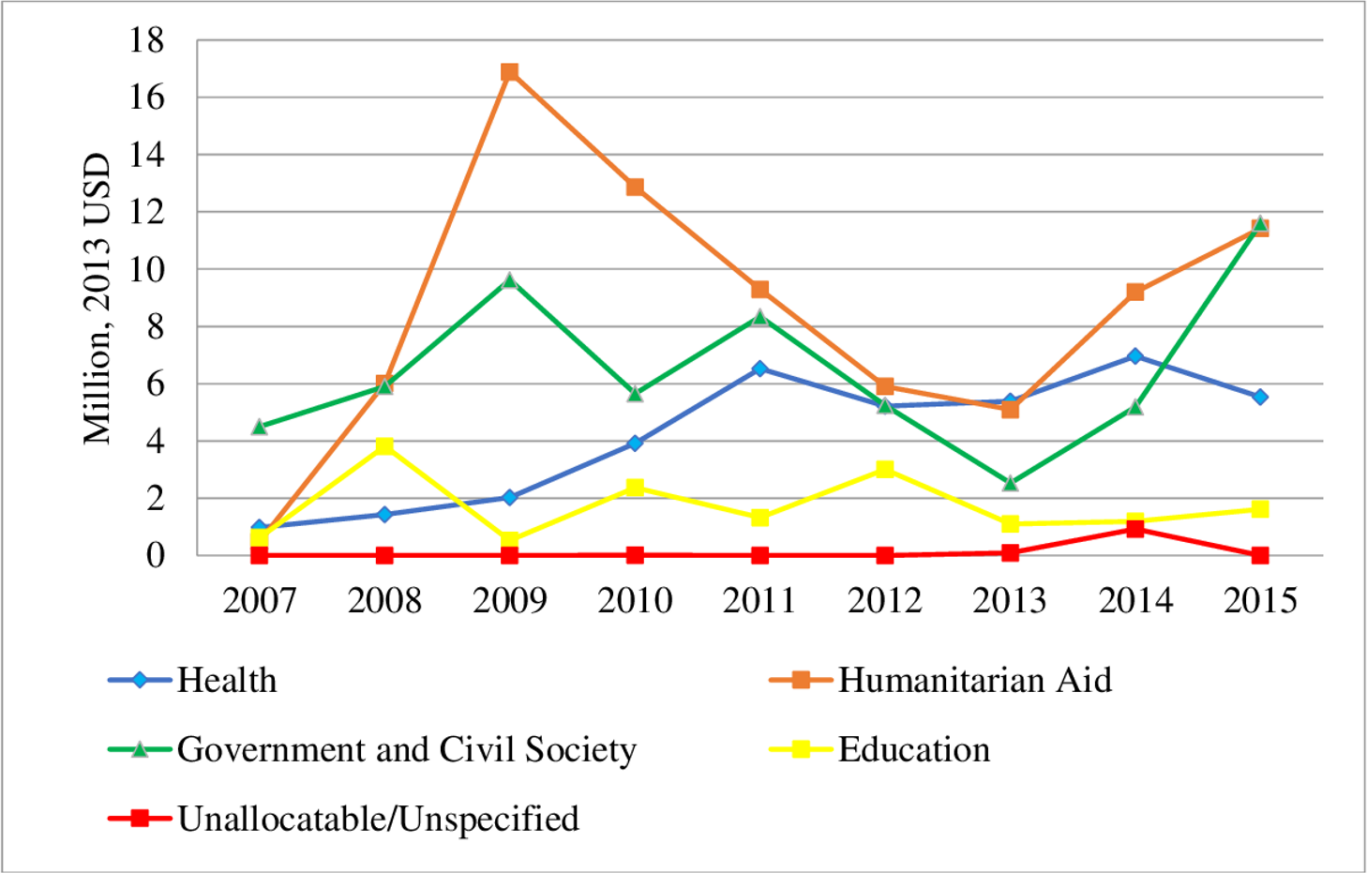
DAMH_CA by year and sector (million, 2013 USD), lower-bound estimates. In this figure, we combined the sectors “General Health” and “Population Program and Reproductive Health” in the original CRS dataset as “Health,” the sectors “Humanitarian Aid” and “Multisector/Crosscutting” as “Humanitarian Aid,” and the sectors “Government and Civil Society” and “Other Social Infrastructure and Services” as “Government and Civil Society.” CRS, Creditor Reporting System; DAMH_CA, development assistance for child and adolescent mental health; USD, US dollar.

The humanitarian aid sector received the largest cumulative DAMH_CA, with a total amount of US$77.2 million (40.5% of total DAMH_CA), followed by the government and civil society sector (US$58.6 million [30.8%]), the health sector (US$38.0 million [20.0%]), and the education sector (US$15.6 million [8.2%]) (**Fig 2**). In terms of each sector’s proportion in DAMH_CA, government and civil services had the largest proportion in 2007 and 2008. It was then replaced by humanitarian assistance between 2009 and 2014 and returned to the leading position in 2015 (38.4% in total DAMH_CA in 2015), followed closely by “Humanitarian Aid” (37.9%), “Health” (18.3%), and “Education” (5.4%).

## DAMH_CA channels and donors, lower-bound estimates

In terms of channel of delivery, from 2007 to 2015, nongovernmental organizations (NGOs) and civil society received the largest cumulative DAMH_CA of US$110.6 million (58.1% of total DAMH_CA), followed by UN organizations and WHO (US$46.4 million [24.4%]); the public sector in recipient countries received a relatively small fraction (US$23.1 million [12.1%]) (see **S2 Fig**).

The top three donors were European Union (EU) institutions (US$44.8 million), Germany (US$21.8 million), and Switzerland (US$18.6 million). During the analysis period, they together provided between 32.1% and 71.7% of total DAMH_CA each year, with a mean of 51.5% (**S3 Fig**). Even for the top 10 donors, their percentage of total DAH on DAMH_CA over the 8 years is pitifully small, from 0.03% in Canada to 2.69% in Finland (**S3 Table**). When using the upper-bound estimates, the Global Fund to Fight AIDS, Tuberculosis and Malaria (GFATM) appeared to be the largest donor (cumulatively US$551.3 million), with most of its investments in providing psychological support and other medical and social care for high-risk populations of HIV/AIDS, including drug users (**S4 Fig**).

## DAMH_CA per capita at the recipient country level, lower-bound estimates

**Fig 3:**
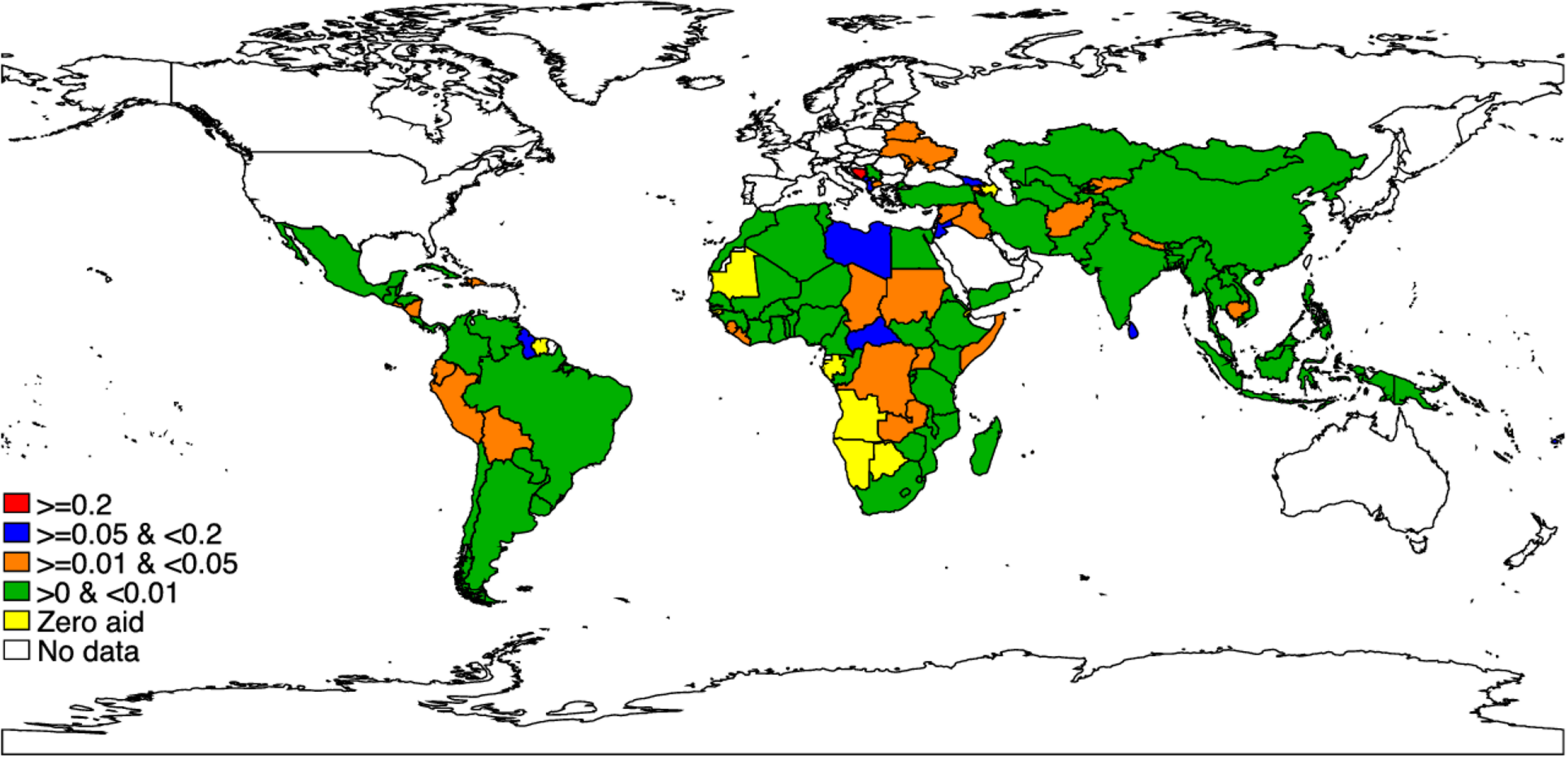
Average annual per capita DAMH_CA disbursement between 2007 and 2015 in each country (2013 USD), lower-bound estimates. (1) The top 10 countries with the highest average annual per capita DAMH_CA disbursement are West Bank and Gaza Strip (US$2.88), Kiribati (US$1.33), Lebanon (US$0.37), Bosnia and Herzegovina (US$0.23), Samoa (US$0.16), Georgia (US$0.15), Jordan (US$0.13), Sri Lanka (US$0.11), Libya (US$0.11), and Central African Republic (US$0.11). (2) We did not include unallocable or regional DAMH_CA projects. DAMH_CA, development assistance for child and adolescent mental health; USD, US dollar.

**Fig 3** presents the world map of average annual per capita DAMH_CA for 132 countries. Throughout the analysis period, 21 countries did not receive any funding on DAMH_CA, including 3 low-income countries (Guinea-Bissau, Gambia, and Comoros), 8 lower middle–income countries (e.g., Lesotho, Tonga, and Djibouti), and 10 upper middle–income countries (e.g., Gabon, Namibia, and Botswana). Per capita, 69 countries received less than US$0.01 DAMH_CA, including 20 low-income countries (e.g. Rwanda, Mozambique, and Guinea), 26 lower middle–income countries (e.g., India, Senegal, and Nigeria), and 23 upper middle–income countries (e.g., China, Mexico, and Chile). Only 14 countries received more than US$0.05 DAMH_CA per capita, with 4 of them receiving more than US$0.2 per capita: West Bank and Gaza Strip (US$2.9 per capita), Kiribati (US$1.3), Lebanon (US$0.4), and Bosnia and Herzegovina (US$0.2). Numerical values of annual DAMH_CA per capita for each country (in both upper and lower bounds) are reported in **S4 Table** and **S5 Table**.

In different regions, projects that received a large amount of DAMH_CA often had different targets. For example, in the Eastern Mediterranean and Europe, DAMH_CA projects with a large amount of disbursements usually focused on alcohol addiction. DAMH_CA projects in Middle Eastern countries (e.g., West Bank and Gaza Strip, Lebanon) were usually focused on coping with trauma and providing psychological support to children and adolescents in conflicts. In Africa, a large amount of DAMH_CA was related to HIV/AIDS projects, which included components addressing drug addiction and psychological care for children and adolescents living with HIV.

## DAMH_CA to suicide and five mental disorders, lower-bound estimates

Interventions targeting the five leading causes for disability or death among children and adolescents received relatively small disbursements. The lower-bound estimates show that the cumulative amount of disbursements to trauma-related mental disorders accounted for 1.13% of total DAMH_CA, followed by substance abuse (0.76%), autism (0.19%), suicide (0.02%), depression (0.02%), and anxiety (0.01%) (see **S9 Fig**). Findings on investments in these disorders remain similar when using upper-bound estimates **(S5 Fig)**.

## Discussion

Using the CRS data with a multi-sectoral perspective, we tracked 44 donors’ aid disbursements to DAMH_CA projects implemented in 132 developing countries between 2007 and 2015. The total amount of DAMH_CA with a primary target on the mental health of children and adolescents was US$190.3 million over the 8 years, accounting for 12.5% of total DAMH and 0.1% of total DAH. Per capita, 90 developing countries received either 0 or less than US$0.01 average DAMH_CA, including 23 low-income countries (out of 36 total low-income countries [64%]). Global child and adolescent mental health is truly the orphan of DAH. Our findings are consistent with a recent publication on DAMH_CA [13].

Additional concerns we observed are related to volatility and poor targeting of assistance. DAMH_CA experienced marked fluctuations over the 8-year period, mainly driven by the investments from two donors. This poses challenges for effective long-term budgeting and planning for recipient countries with high dependence on external sources for mental health financing. Furthermore, it is alarming that both the total amount and proportion of DAMH_CA for the public sectors has been comparatively very low (12.1% over the 8-year period), with most DAMH_CA targeting temporary or short-term humanitarian assistance to children and adolescents in disasters or conflicts and channeling the assistance through NGOs. This is consistent with our previous study on DAMH, in which we also observed that the share of DAMH channeled to public health sectors, the key player in delivering basic mental health care, was small. Though there is mounting evidence that mental health services for children and adolescents can be effectively delivered by community health workers [14], only a tiny fraction of aid has been invested (US$14.2 million over the 8-year period) in delivering mental health care to children and adolescents with priority mental health conditions.

This analysis has the following limitations. (1) Due to a lack of details of aid projects at the recipient country level, our estimates did not include aid to developing countries from emerging economies (e.g., China), NGOs, or foundations (with the exception of the BMGF). (2) The identification strategy was not able to capture all projects on DAMH_CA or those with typographical errors and solely relied on project descriptions provided by donors, which could be subject to unreliable quality. (3) When estimating DAMH_CA at the country level, data that could not be allocated to a specific recipient country were not included. (4) The estimates of DAMH_CA for the specific mental disorders (e.g., depression) in the analysis could be underestimated, as projects with general mental health terms as key words (e.g., projects of “mental health”) could also provide care to patients with depression but not be included in the current estimates due to lack of information regarding separate funds for depression. (5) Our estimation only focused on projects that either primarily or partially targeted mental health for children and adolescents. We did not include projects that targeted socioeconomic determinants of child and adolescent mental health, such as nutrition or maternal education; thus, our estimates may largely reflect investments in selective prevention (e.g., children in humanitarian contexts) or treatment and care.

Despite these limitations, this analysis is the first to report on the financing of mental health care for children and adolescents in low- and middle-income countries (LMICs) between 2007 and 2015. We consider a number of key lessons to be drawn from this analysis. First, given the evidence that much of the burden of mental disorders has its origins in childhood or adolescence and early interventions are potentially the key to prevention and recovery [15,16], there should be an increase, at least a doubling, in DAMH_CA so as to enable developing countries to strengthen their capacity by investing in mental health infrastructure and training providers, including community health workers and specialists, to deliver cost-effective interventions. We believe that challenging the stigma associated with mental disorders, providing accurate information about the prevalence and impact of mental disorders in children and adolescents, and cost-effectiveness of interventions will raise interest in investing in mental health among the donor community. Second, investments should be based on scientific evidence of the patterns of the burden of disease, cost-effective interventions, and a recipient country’s own sociocultural context. For example, as community-based delivery of psychosocial interventions by frontline workers has been shown to be cost-effective in LMICs, more funds should be directed to this approach. Third, while it is important for donors to continue supporting humanitarian assistance for those living in conflict areas, there should be more donor investments, with more stability and better targeting for building and sustaining mental health programs to achieve universal mental health coverage in the long term. In particular, funding should target the public system to ensure that the goals of integrating mental health interventions (promotional, preventive, or curative) into appropriate platforms for delivery, including health, education, and social sectors, are achieved [17].

## Supporting information

S1 Text Data sources and identification strategy. (DOCX)

S1 Table One hundred thirty-two recipients in the CRS (according to the World Bank income classification in 2010). CRS, Creditor Reporting System.
(XLSX)

S2 Table Key words used to search DAMH_CA projects in the CRS, 2007–2015. CRS, Creditor Reporting System; DAMH_CA, development assistance for child and adolescent mental health.
(XLSX)

S3 Table Top 10 DAMH_CA donors: DAMH_CA (lower bound) as a percentage of their DAH over the 8 years. DAH, development assistance for health; DAMH_CA, development assistance for child and adolescent mental health.
(XLSX)

S4 Table Annual DAMH_CA disbursements per capita (2013 USD) to each recipient country between 2007 and 2015, lower-bound estimates. DAMH_CA, development assistance for child and adolescent mental health; USD, US dollar.
(XLSX)

S5 Table Annual DAMH_CA disbursements per capita (2013 USD) to each recipient country between 2007 and 2015, upper-bound estimates. DAMH_CA, development assistance for child and adolescent mental health; USD, US dollar.
(XLSX)

S1 Box Definition of sectors in CRS data. CRS, Creditor Reporting System.
(DOCX)

S1 Fig Annual proportion of DAMH_CA in total DAH between 2007 and 2015, lower-bound estimates. DAH, development assistance for health; DAMH_CA, development assistance for child and adolescent mental health.
(TIFF)

S2 Fig Annual DAMH_CA (million, 2013 USD) by channel between 2007 and 2015, lower-bound estimates. UN organizations include UN, UNFPA, UNICEF, UNDP, UNAIDS, and UNECE. DAMH_CA, development assistance for child and adolescent mental health; EU, European Union; UN, United Nations; UNAIDS, the Joint United Nations Programme on HIV and AIDS; UNDP, United Nations Development Programme; UNECE: United Nations Economic Commission for Europe; UNFPA, United Nations Population Fund; UNICEF, United Nations Children’s Fund; USD, US dollar.
(TIFF)

S3 Fig Annual DAMH_CA (million, 2013 USD) by donor between 2007 and 2015, lower-bound estimates. DAMH_CA, development assistance for child and adolescent mental health; USD, US dollar.
(TIFF)

S4 Fig Annual DAMH_CA (million, 2013 USD) by donor between 2007 and 2015, upper-bound estimates. DAMH_CA, development assistance for child and adolescent mental health; USD, US dollar.
(TIFF)

S5 Fig Cumulative DAMH_CA disbursement (million, 2013 USD) of suicide and mental disorders and their percentages in total DAMH_CA between 2007 and 2015, lower-bound estimates. The DAH to each health focus shown in this figure is not mutually exclusive. For example, if a project is for both anxiety and depression, we included this project in the estimation of both health focuses. DAH, development assistance for health; DAMH_CA, development assistance for child and adolescent mental health; USD, US dollar.
(TIFF)

S6 Fig DAMH_CA by year (million, 2013 USD) and DAMH_CA disbursements as percentages of total DAMH between 2007 and 2015, upper-bound estimates. DAMH, development assistance for mental health; DAMH_CA, development assistance for child and adolescent mental health; USD, US dollar.
(TIFF)

S7 Fig Annual DAMH_CA (million, 2013 USD) by sector (combined) between 2007 and 2015, upper-bound estimates. In this figure, we combined the sectors of “General Health” and “Population Program and Reproductive Health” in the original CRS dataset as “Health,” the sectors of “Humanitarian Aid” and “Multisector/Crosscutting” as “Humanitarian Aid,” and the sectors of “Government and Civil Society” and “Other Social Infrastructure and Services” as “Government and Civil Society.” CRS, Creditor Reporting System; DAMH_CA, development assistance for child and adolescent mental health; USD, US dollar.
(TIFF)

S8 Fig Annual DAMH_CA (million, 2013 USD) by channel between 2007 and 2015, upper-bound estimates. UN organizations include UN, UNFPA, UNICEF, UNDP, UNAIDS, and UNECE. DAMH_CA, development assistance for child and adolescent mental health; EU, European Union; UN; United Nations; UNAIDS, the Joint United Nations Programme on HIV and AIDS; UNDP, United Nations Development Programme; UNECE, United Nations Economic Commission for Europe; UNFPA; United Nations Population Fund; UNICEF, United Nations Children’s Fund; USD, US dollar.
(TIFF)

S9 Fig Per capita DAMH_CA disbursement (2013 USD) between 2007 and 2015 in each country, upper-bound estimates. We did not include unallocable or regional DAMH_CA projects. DAMH_CA, development assistance for child and adolescent mental health; USD, US dollar.
(TIF)

S10 Fig Cumulative DAMH_CA disbursement (million, 2013 USD) between 2007 and 2015 by health focus, upper-bound estimates. DAMH_CA, development assistance for child and adolescent mental health; USD, US dollar.
(TIF)

## Abbreviations

BMGF: Bill & Melinda Gates Foundation
CRS: Creditor Reporting System
DAC: Development Assistance Committee
DAH: development assistance for health
DALY: disability-adjusted life year
DAMH: development assistance for mental health
DAMH_CA: development assistance for child and adolescent mental health
EU: European Union
GFATM: Global Fund to Fight AIDS, Tuberculosis and Malaria
LMIC: low- and middle-income country
NGO: nongovernmental organization
OECD: Organization for Economic Co-operation and Development
USD: US dollar
