# Supplementary material for: Global child and adolescent mental health: The orphan of development assistance for health
Source: PLoS Med. 2018 Mar 9;15(3):e1002524. doi: 10.1371/journal.pmed.1002524 (PMC5844520; doi:10.1371/journal.pmed.1002524)
Supplement: S1 Text — (DOCX) [file pmed.1002524.s001.docx]

**S1 Text. Data sources and Identification Strategy**

*Data sources*

The CRS has been commonly used in tracking aid to health-related projects.[1-4] The data includes projects implemented in 148 developing countries, classified by the World Bank.[5] We excluded 16 states or territories without a complete time series data on child/adolescent populations. The final sample has 36 low-income countries, 53 low-middle income countries, and 43 upper-middle income countries. Annual data on child and adolescent populations is derived from data provided by the United Nations Population Division (UNPD).[6] Since the UNPD data by age group was reported every five years, we imputed the years in between assuming the population was growing at the same rate in each year.

*Identifying projects on child and adolescent mental health*

The CRS data does not have a variable to indicate mental health projects for children and adolescent, but has three variables (project title, short description of a project, and long description of a project) that allowed us to identify such projects via a list of keywords. The keywords were constructed based on those words used in the previous studies.[1,7] The keywords contain two parts: one part was used to identify projects on mental health (e.g. “mental”, “depression”, “autism”), and the other part was used to identify age of the targeted populations (e.g. “child”, “adolescent”, “school-age”) (**S2 Table**). A project was identified as for DAMH_CA if it contained keywords from the both parts.

When searching for projects with the keywords, we followed previous practices [1,2,4] and used a combination of keyword searches and manual review, with keyword search as the first step and manually reviewing projects which survived the keywords search as the second step. Our strategy may lead to missing some projects for DAMH_CA due to imperfect sensitivity of the keyword search. To investigate how many projects for DAMH_CA were not captured, we randomly selected 10% of the projects from the health sector data in 2011 and applied both keywords search and manual coding separately to the selected projects. We compared the results derived from these two methods and found that the keyword search approach missed about 0.8% of projects for DAMH_CA. We investigated how many projects that were not related to DAMH_CA were falsely coded as DAMH_CA projects when using the keyword search. We exported the data of three variables (project title, short description of the project, and long description of the project) into an excel sheet and reviewed each entry to identify the projects that were falsely coded as DAMH_CA. We found that 21% of the projects were falsely coded as DAMH and excluded them from the analysis. We kept 4,009 projects for further analyses.

*Constructing upper-bound and lower-bound estimates*

In the CRS data, a project may have multiple targets, only some of which are related to mental health. Using the three variables (project title, short description of the project, and long description of the project), we were able to identify if a project has mental health as its primary focus or a project has multiple targets including mental health (for example, a project targeting teenagers’ nutrition and mental health). For projects with multiple targets, the CRS does not have information which allows us to identify the amount of aid attached to each of these targets in a project. We therefore followed the approach from an earlier study [2] and constructed two sets of estimates: one including the full disbursements of multi-target projects (the upper bound of estimated aid for DAMH_CA) and primary-target projects, and the other (the lower bound of DAMH_CA) which only included projects with primary target on promoting mental health for children and adolescents.

Reference:

1. Gilbert BJ, Patel V, Farmer PE, Lu C. Assessing Development Assistance for Mental Health in Developing Countries: 2007–2013. PLOS Med. 2015 Jun 2;12(6):e1001834.

2. Lu C, Chu A, Li Z, Shen J, Subramanian S, Hill K. Assessing development assistance for child survival between 2000 and 2014: A multi-sectoral perspective. Fan VY, editor. PLoS One. 2017 Jul 11;12(7):e0178887.

3. Lu C, Schneider MT, Gubbins P, Leach-Kemon K, Jamison D, Murray CJ. Public financing of health in developing countries: a cross-national systematic analysis. Lancet. 2010 Apr;375(9723):1375–87.

4. Pitt C, Lawn JE, Ranganathan M, Mills A, Hanson K. Donor Funding for Newborn Survival: An Analysis of Donor-Reported Data, 2002–2010. Stuckler D, editor. PLoS Med. 2012 Oct 30;9(10):e1001332.

5. The World Bank. World Bank Country and Lending Groups – World Bank Data Help Desk [Internet]. [cited 2017 Oct 4]. Available from: https://datahelpdesk.worldbank.org/knowledgebase/articles/906519-world-bank-country-and-lending-groups

6. UNDP. World Population Prospects - Population Division - United Nations [Internet]. [cited 2017 Aug 2]. Available from: https://esa.un.org/unpd/wpp/Download/Standard/Population/

7. Charlson FJ, Dieleman J, Singh L, Whiteford HA. Donor Financing of Global Mental Health, 1995—2015: An Assessment of Trends, Channels, and Alignment with the Disease Burden. Maulik PK, editor. PLoS One. 2017 Jan 3;12(1):e0169384.
