# Supplementary material for: Global child and adolescent mental health: The orphan of development assistance for health
Source: PLoS Med. 2018 Mar 9;15(3):e1002524. doi: 10.1371/journal.pmed.1002524 (PMC5844520; doi:10.1371/journal.pmed.1002524)
Supplement: S1 Box — CRS, Creditor Reporting System. (DOCX) [file pmed.1002524.s007.docx]

**Box 1 Definition of sectors in CRS data**

(data source: http://www.oecd.org/dac/stats/purposecodessectorclassification.htm)

**Education**

Basic Education: primary education, basic life skills for youth and adults, early childhood education

Secondary Education: secondary education and vocational training

Post-Secondary Education: high education, advanced technical and managerial training

Education, Level Unspecified: educational policy and administrative management, education facilities and training, teacher training, educational research

**Health**

General Health: health policy and administrative management, medical education/training, medical research, medical services

Basic Health: basic health care, basic health infrastructure, basic nutrition, infectious disease control, health education, malaria control, tuberculosis control, health personnel development

Population and Reproductive Health: population policy and administrative management, reproductive health care, family planning, STD control including HIV/AIDS, personnel development

**Government and Civil Service**

Government & Civil Society: public sector policy and administrative management, public finance management, decentralization, anti-corruption, legal and judicial development, human rights, women’s equality, media freedom, democratic participation and civil society, elections, legislatures

Conflict, Peace & Security: security system management and reform, civilization peace-building, conflict prevention and resolution, international peacekeeping operations, child solider, removal of land mines and explosive remnants of war, reintegration and small arms and light weapons

**Other Social Infrastructure & Services**

Social/welfare services, housing policy, administrative management, basic social services, culture and recreation, statistical capacity building, narcotics control, social mitigation of HIV/AIDS

**Humanitarian aid**

Emergency response: food aid, material relief assistance and relief, protection and support services

Reconstruction Relief and Rehabilitation

Disaster Prevention & Preparedness

**Other Multisector**

Rrural and urban development, multisector education/training, research and scientific institutions, environmental policy, education/training, and administrative management

**Unallocated / Unspecified**

Sectors not specified, promotion of development awareness in donor country
