# Supplementary figures and images for: Global child and adolescent mental health: The orphan of development assistance for health
Source: PLoS Med. 2018 Mar 9;15(3):e1002524. doi: 10.1371/journal.pmed.1002524 (PMC5844520; doi:10.1371/journal.pmed.1002524)

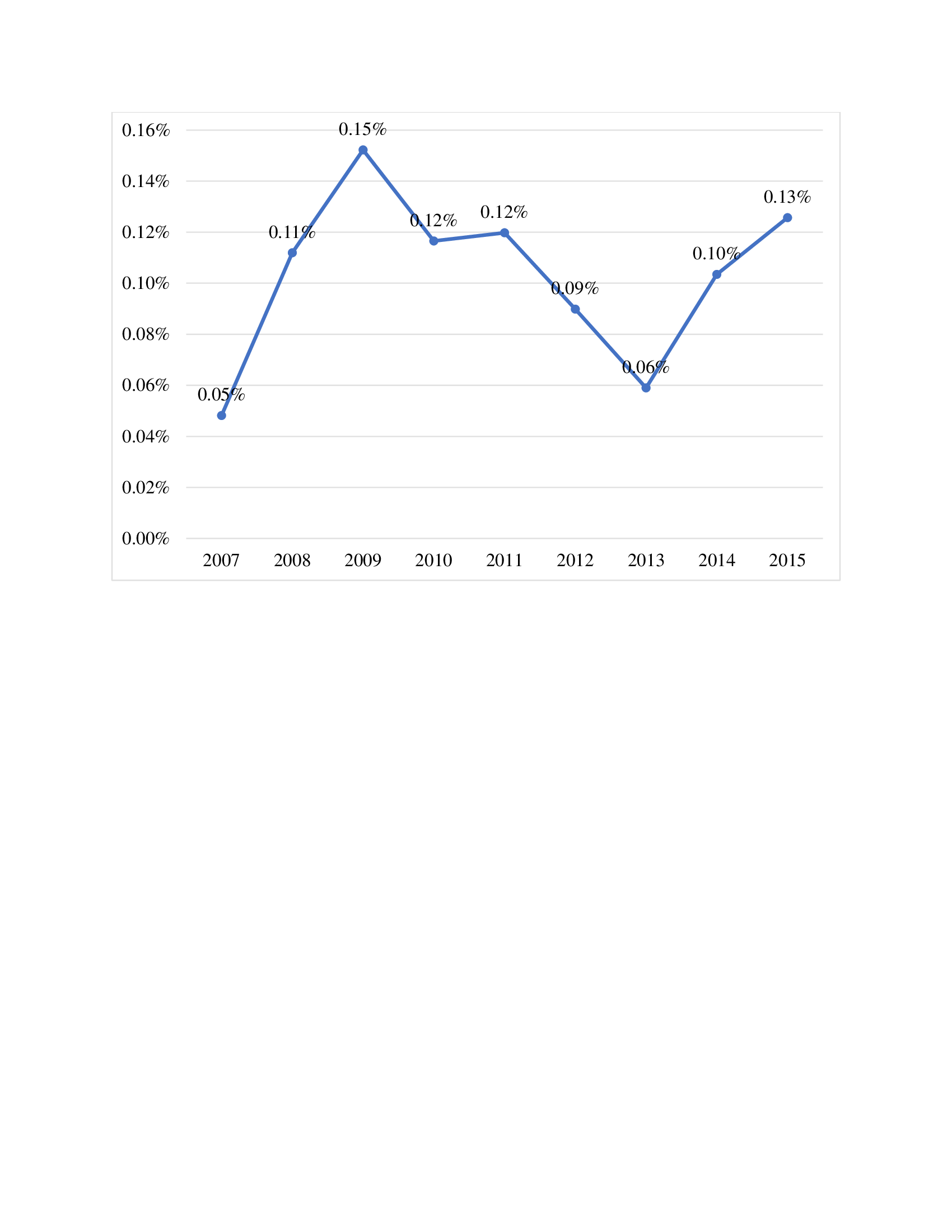

Supplement: S1 Fig — DAH, development assistance for health; DAMH_CA, development assistance for child and adolescent mental health. (TIFF) [file pmed.1002524.s008.tiff]

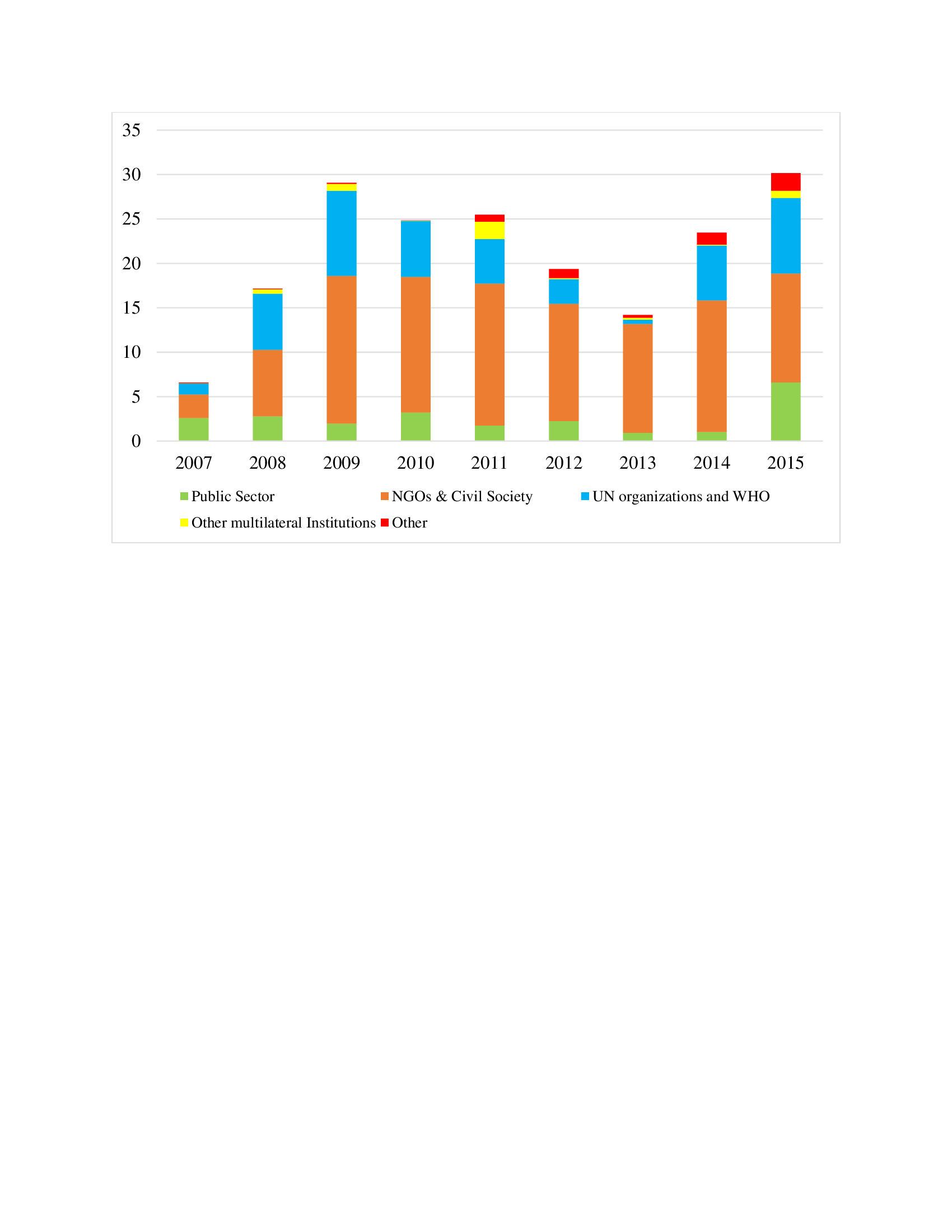

Supplement: S2 Fig — UN organizations include UN, UNFPA, UNICEF, UNDP, UNAIDS, and UNECE. DAMH_CA, development assistance for child and adolescent mental health; EU, European Union; UN, United Nations; UNAIDS, the Joint United Nations Programme on HIV and AIDS; UNDP, United Nations Development Programme; UNECE: United Nations Economic Commission for Europe; UNFPA, United Nations Population Fund; UNICEF, United Nations Children’s Fund; USD, US dollar. (TIFF) [file pmed.1002524.s009.tiff]

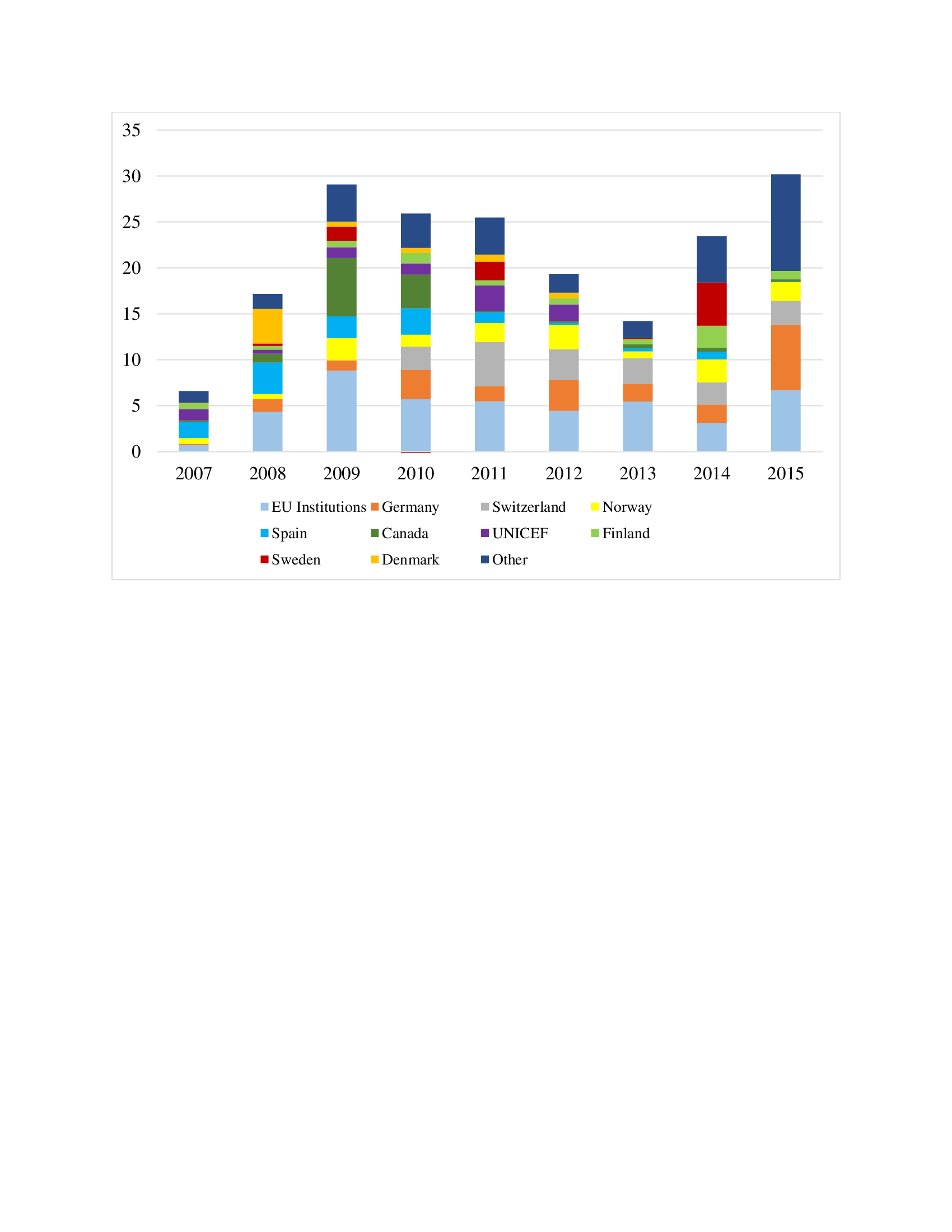

Supplement: S3 Fig — DAMH_CA, development assistance for child and adolescent mental health; USD, US dollar. (TIFF) [file pmed.1002524.s010.tiff]

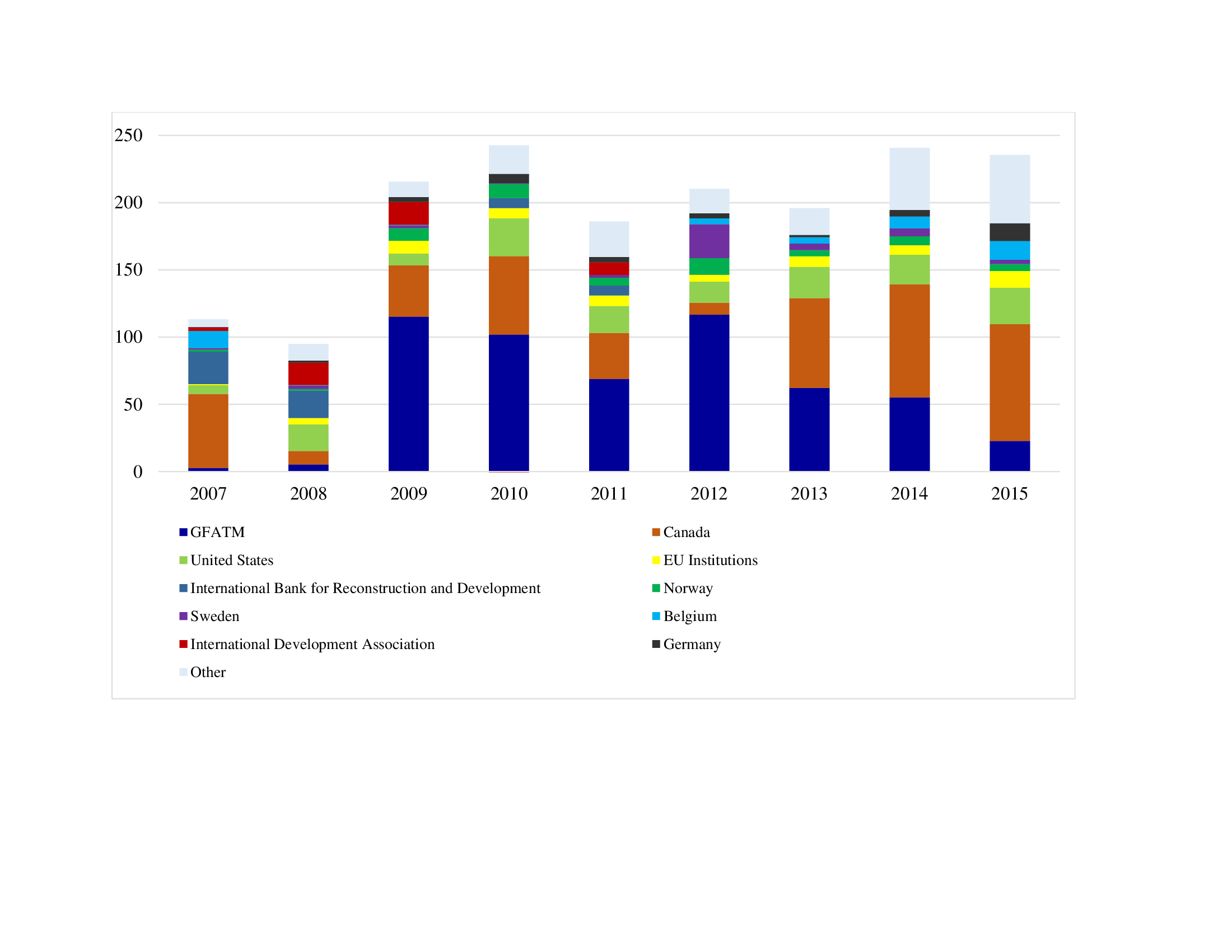

Supplement: S4 Fig — DAMH_CA, development assistance for child and adolescent mental health; USD, US dollar. (TIFF) [file pmed.1002524.s011.tiff]

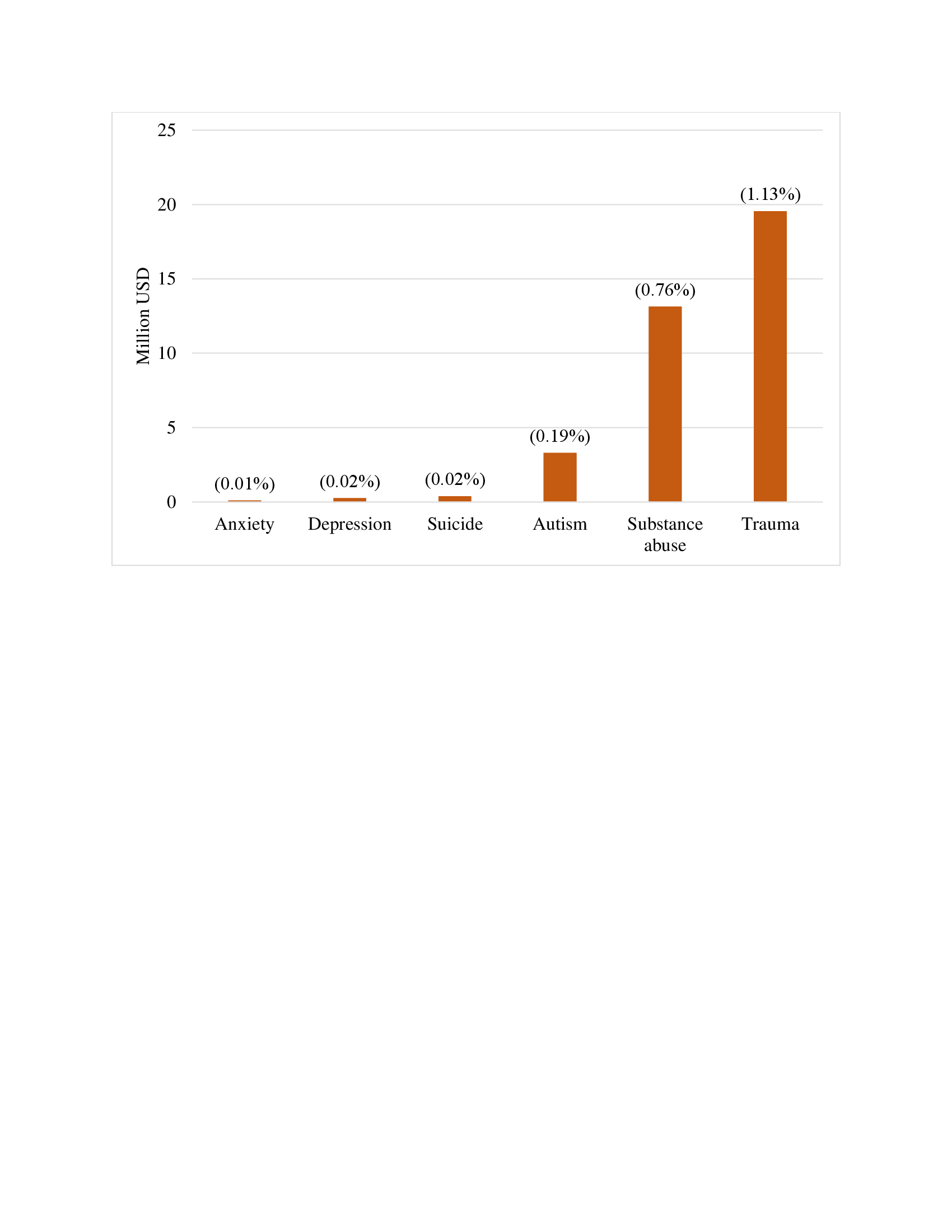

Supplement: S5 Fig — The DAH to each health focus shown in this figure is not mutually exclusive. For example, if a project is for both anxiety and depression, we included this project in the estimation of both health focuses. DAH, development assistance for health; DAMH_CA, development assistance for child and adolescent mental health; USD, US dollar. (TIFF) [file pmed.1002524.s012.tiff]

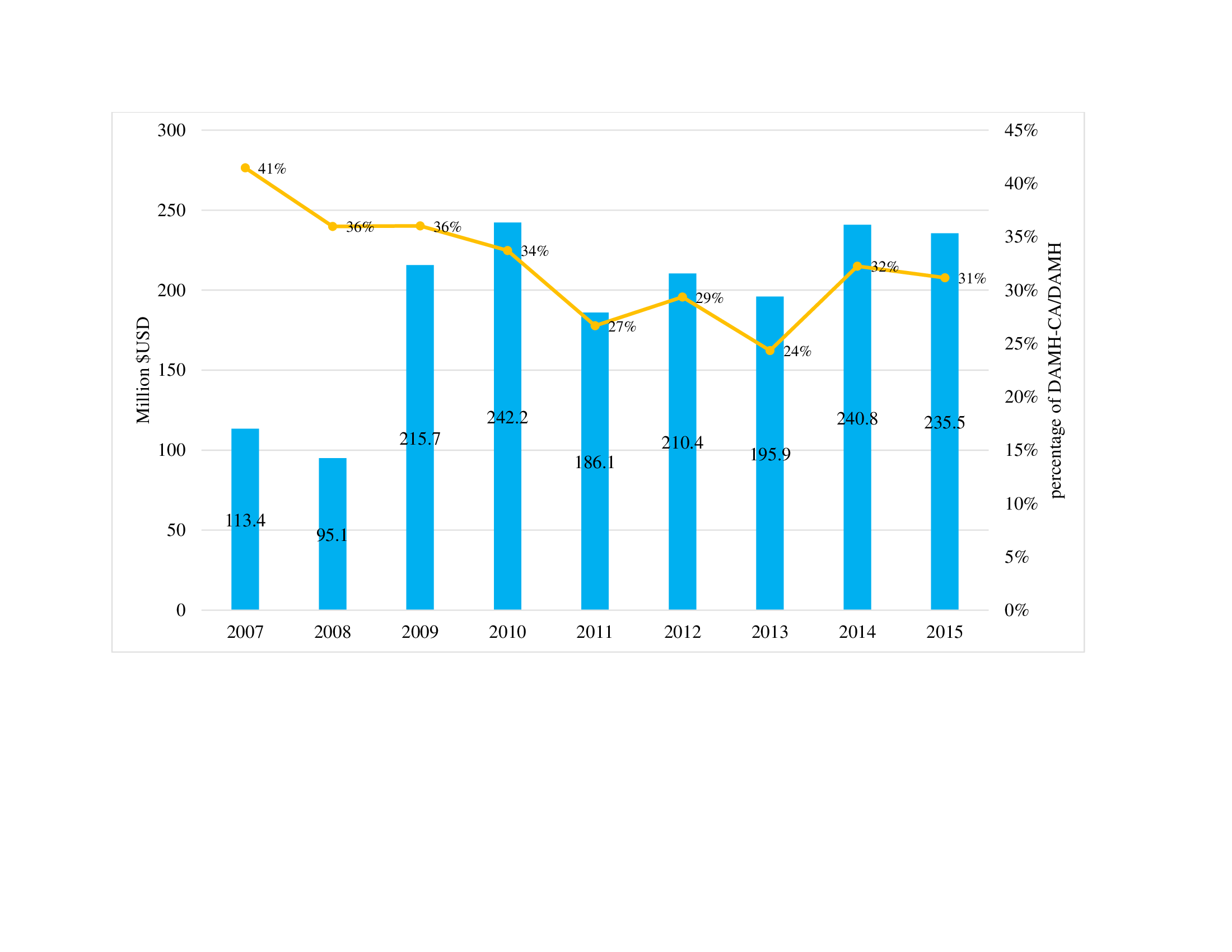

Supplement: S6 Fig — DAMH, development assistance for mental health; DAMH_CA, development assistance for child and adolescent mental health; USD, US dollar. (TIFF) [file pmed.1002524.s013.tiff]

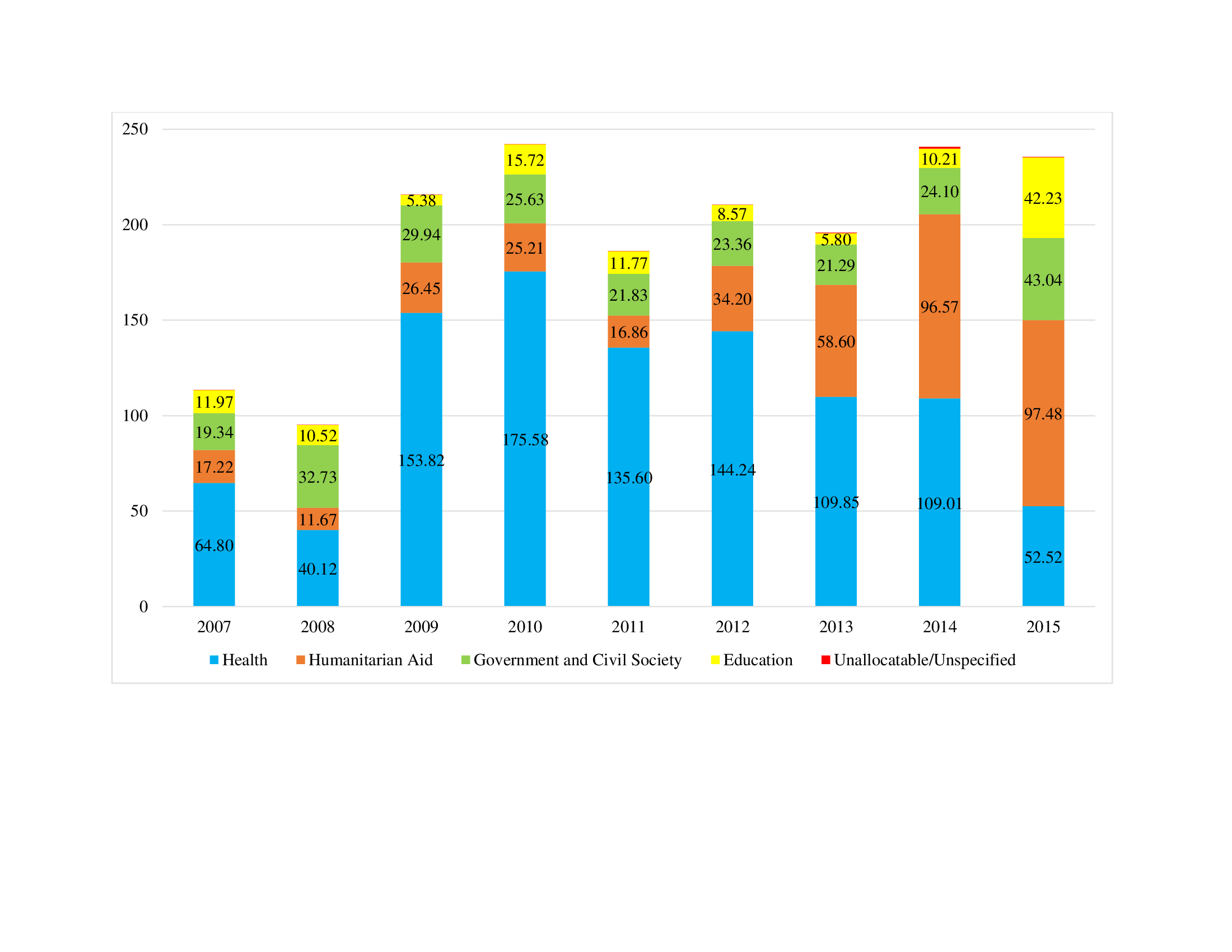

Supplement: S7 Fig — In this figure, we combined the sectors of “General Health” and “Population Program and Reproductive Health” in the original CRS dataset as “Health,” the sectors of “Humanitarian Aid” and “Multisector/Crosscutting” as “Humanitarian Aid,” and the sectors of “Government and Civil Society” and “Other Social Infrastructure and Services” as “Government and Civil Society.” CRS, Creditor Reporting System; DAMH_CA, development assistance for child and adolescent mental health; USD, US dollar. (TIFF) [file pmed.1002524.s014.tiff]

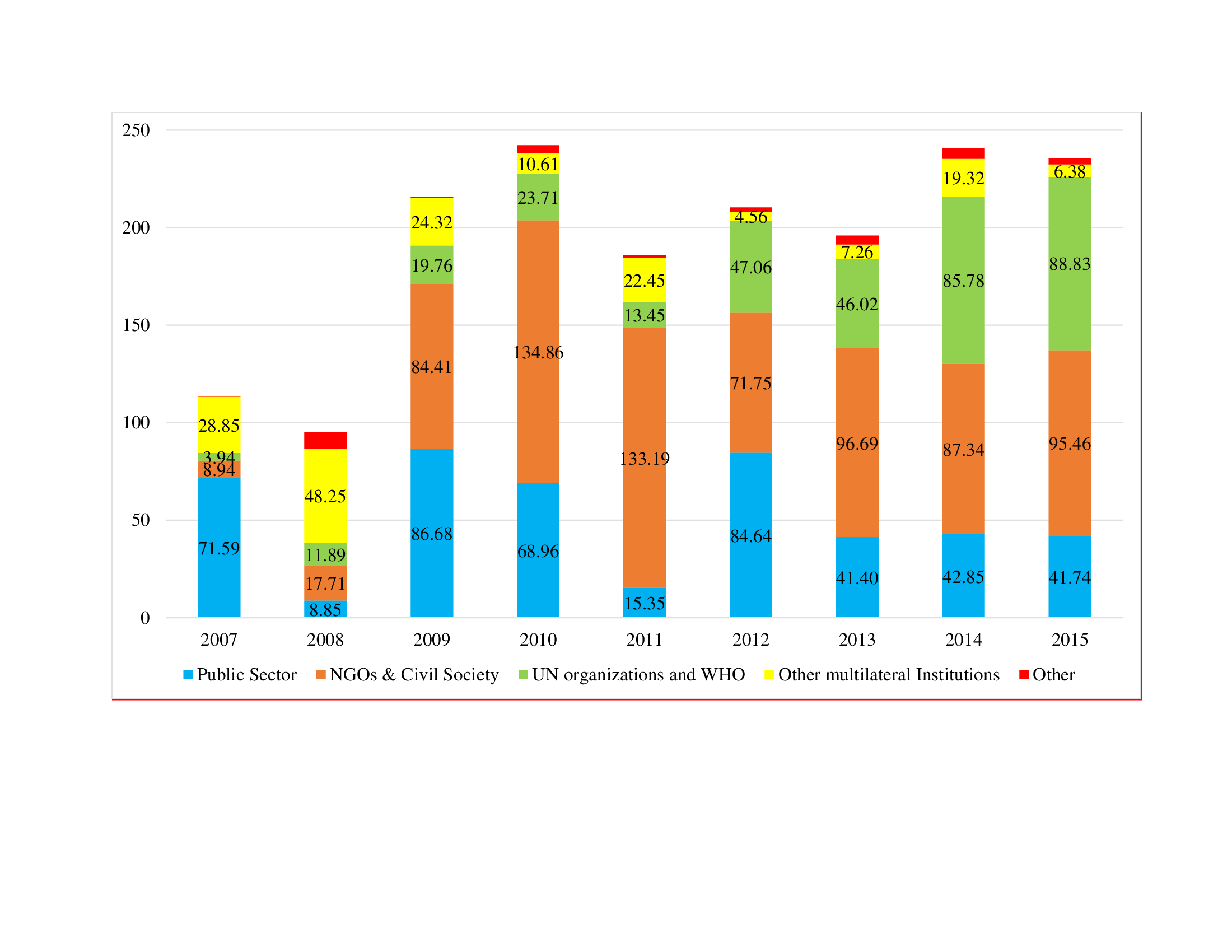

Supplement: S8 Fig — UN organizations include UN, UNFPA, UNICEF, UNDP, UNAIDS, and UNECE. DAMH_CA, development assistance for child and adolescent mental health; EU, European Union; UN; United Nations; UNAIDS, the Joint United Nations Programme on HIV and AIDS; UNDP, United Nations Development Programme; UNECE, United Nations Economic Commission for Europe; UNFPA; United Nations Population Fund; UNICEF, United Nations Children’s Fund; USD, US dollar. (TIFF) [file pmed.1002524.s015.tiff]

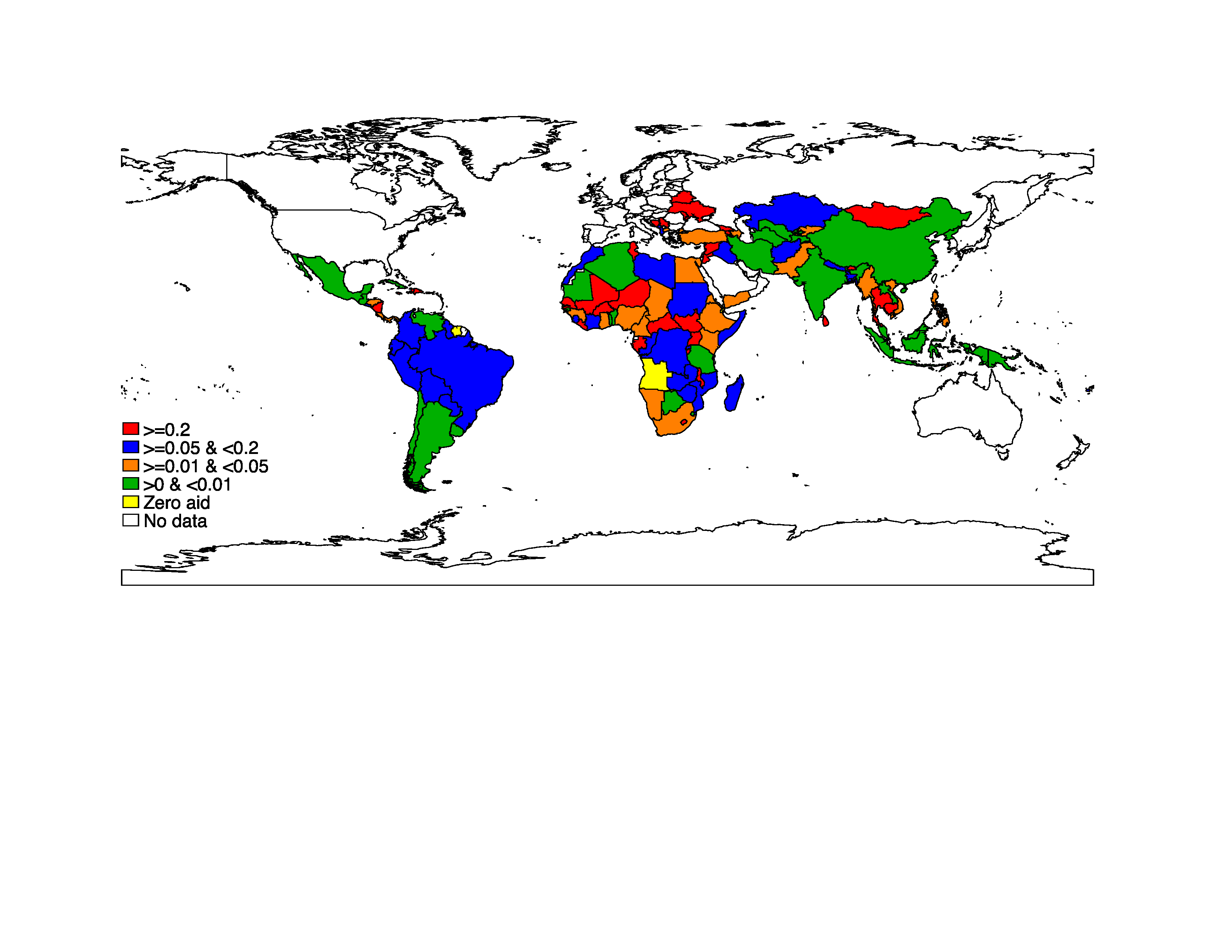

Supplement: S9 Fig — We did not include unallocable or regional DAMH_CA projects. DAMH_CA, development assistance for child and adolescent mental health; USD, US dollar. (TIF) [file pmed.1002524.s016.tif]

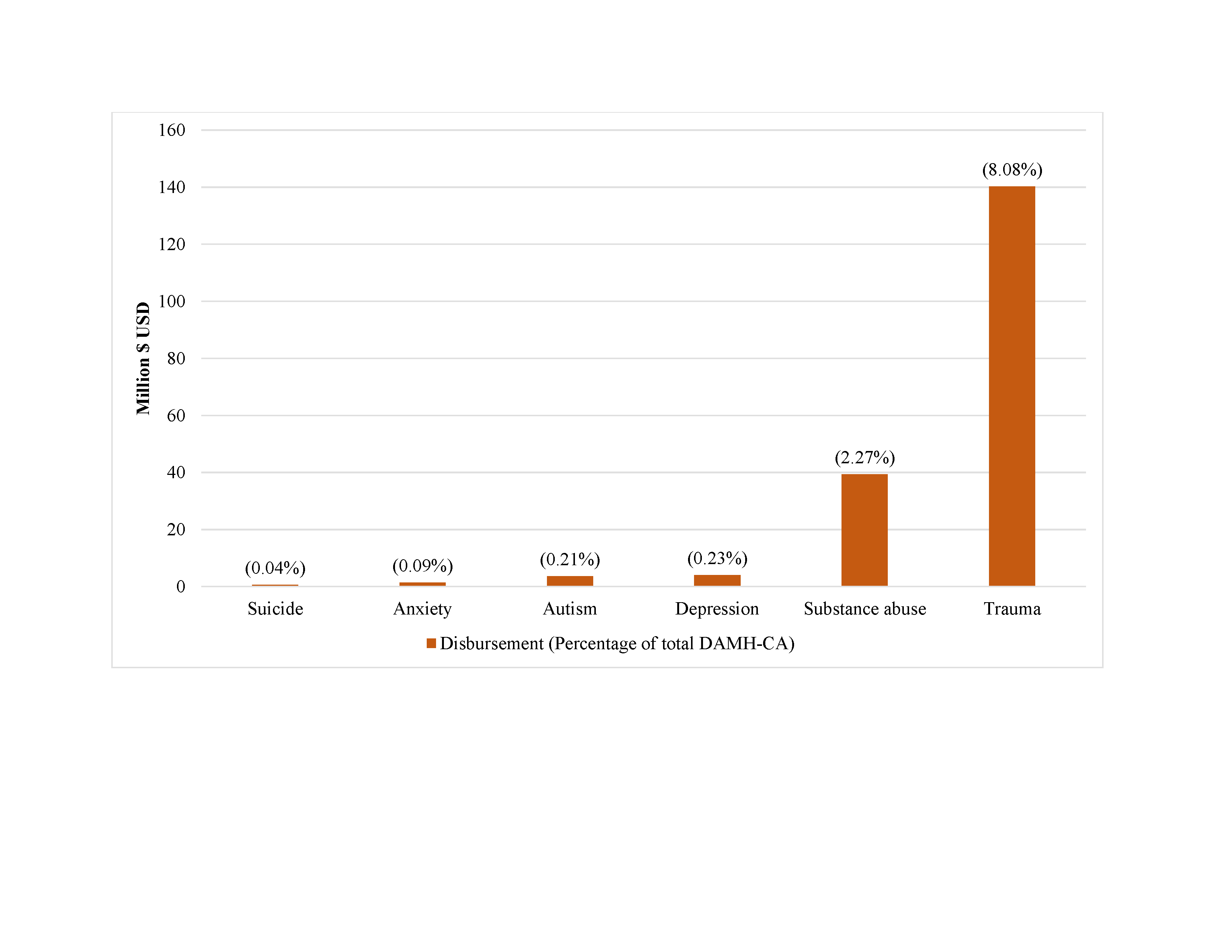

Supplement: S10 Fig — DAMH_CA, development assistance for child and adolescent mental health; USD, US dollar. (TIF) [file pmed.1002524.s017.tif]
